# Prophylactic Chronic Zinc Administration Increases Neuroinflammation in a Hypoxia-Ischemia Model

**DOI:** 10.1155/2016/4039837

**Published:** 2016-08-18

**Authors:** Constantino Tomas-Sanchez, Victor Manuel Blanco-Alvarez, Juan Antonio Gonzalez-Barrios, Daniel Martinez-Fong, Guadalupe Garcia-Robles, Guadalupe Soto-Rodriguez, Eduardo Brambila, Maricela Torres-Soto, Alejandro Gonzalez-Vazquez, Ana Karina Aguilar-Peralta, José-Luis Garate-Morales, Luis-Angel Aguilar-Carrasco, Daniel I. Limón, Jorge Cebada, Bertha Alicia Leon-Chavez

**Affiliations:** ^1^Facultad de Ciencias Químicas, Benemérita Universidad Autonoma de Puebla, 14 Sur y Avenida San Claudio, 72570 Puebla, PUE, Mexico; ^2^Laboratorio de Medicina Genómica, Hospital Regional 1° de Octubre, ISSSTE, Avenida Instituto Politécnico Nacional, No. 1669, 07760 Ciudad de México, Mexico; ^3^Departamento de Fisiología, Biofísica y Neurociencias, Centro de Investigación y de Estudios Avanzados del Instituto Politécnico Nacional, Apartado Postal 14-740, 07000 Ciudad de México, Mexico; ^4^Facultad de Medicina, Benemérita Universidad Autonoma de Puebla, 13 Sur 3101, 72420 Puebla, PUE, Mexico

## Abstract

Acute and subacute administration of zinc exert neuroprotective effects in hypoxia-ischemia animal models; yet the effect of chronic administration of zinc still remains unknown. We addressed this issue by injecting zinc at a tolerable dose (0.5 mg/kg weight, i.p.) for 14 days before common carotid artery occlusion (CCAO) in a rat. After CCAO, the level of zinc was measured by atomic absorption spectrophotometry, nitrites were determined by Griess method, lipoperoxidation was measured by Gerard-Monnier assay, and mRNA expression of 84 genes coding for cytokines, chemokines, and their receptors was measured by qRT-PCR, whereas nitrotyrosine, chemokines, and their receptors were assessed by ELISA and histopathological changes in the temporoparietal cortex-hippocampus at different time points. Long-term memory was evaluated using Morris water maze. Following CCAO, a significant increase in nitrosative stress, inflammatory chemokines/receptors, and cell death was observed after 8 h, and a 2.5-fold increase in zinc levels was detected after 7 days. Although CXCL12 and FGF2 protein levels were significantly increased, the long-term memory was impaired 12 days after reperfusion in the Zn+CCAO group. Our data suggest that the chronic administration of zinc at tolerable doses causes nitrosative stress, toxic zinc accumulation, and neuroinflammation, which might account for the neuronal death and cerebral dysfunction after CCAO.

## 1. Introduction

Cerebral ischemia is one of the most common types of cerebrovascular diseases and is the third cause of death and disability worldwide [1]. Cerebral ischemia triggers a complex cascade of events at genomic, molecular, and cellular levels that mainly leads to inflammation in the central nervous system (CNS) as well as in the periphery [2]. Neuroinflammation is characterized by microglia migration and leucocyte infiltration into the damaged tissue and by the production of free radicals and immunological mediators [3]. Among the immunological mediators, interleukins and chemokines are significantly increased in patients with cerebral ischemia [4, 5]. These proteins play an important role in neuronal survival, angiogenesis, and recruitment of inflammatory cells and progenitor cells [6].

During brain ischemia, several chemokines are expressed to perform a great diversity of cellular functions. For instance, CXCL1 stimulates chemoattraction of neutrophils into the damaged areas [7]. CCL2 and CX3CL (fractalkine) alter the permeability of the blood brain barrier (BBB), promote migration of both macrophages [8] and bone marrow-derived stem cells into ischemic cerebral areas, and contribute to the regeneration of the injured area [9]. CCL3 and CCL4, which are increased 6 hours after ischemia, recruit lymphocytes, macrophages, and microglia [10, 11]. CCL5 and IL-8 stimulate leukocyte infiltration into the ischemic brain [12]. CXCL12 and its receptor CXCR4 recruit neural stem cells, thus contributing to the recovery of the damaged brain area, and enhance angiogenesis from endothelial progenitor cells [13]. In brain ischemia, growth factors are also expressed in an attempt to counteract the ischemic injury; for instance, insulin-like growth factor 1 (IGF-1) promotes cell survival and neuroregeneration [14], and basic fibroblast growth factor (FGF2) promotes proliferation of progenitor cells and reduces the expression of inflammatory factors [15].

In the last two decades, zinc administration has been considered as a prophylactic agent in systemic inflammation [16, 17], as supported by the finding that zinc deficiency decreases the number of immune cells and alters their functions [18, 19]. However, monocytes and macrophages are more resistant to zinc deficiency and high zinc levels [20]. In the central nervous system (CNS), the effect of zinc administration on neuroinflammation has not yet been completely characterized especially following cerebral ischemia. A study has reported that the superacute administration of zinc protoporphyrin (ZnPP) provides neuroprotective effects by blocking interleukin-1 (IL-1) and cerebral edema [21]. Recently, it has been shown that the subacute administration of zinc before an ischemic process provides significant neuroprotection to hippocampal CA1 layer, thus reducing cell death [22]. This neuroprotection might be mediated by CCL2 and its receptor CCR2 [23] and IGF-1, FGF2, and vascular endothelial growth factor (VEGF) [23–25]. Interestingly, all these growth factors are involved in cell survival, proliferation, migration, and maturity of neuronal cells after a brain lesion [26]. Zinc also stabilizes the structure of nerve growth factor (NGF) and regulates the NGF-activated signaling pathway [27].

Zinc is highly localized in the synaptic vesicle of mossy fibers of the dentate granule cells [28, 29], an area highly involved in neuroplasticity in adulthood [30]. Two opposite experiments support the role of zinc in neuroplasticity. Zinc deficiency on the one hand decreased the performance in a short-term-memory task in rodents [31], whereas the supplementation of zinc during the pre- and postnatal periods significantly improved memory in rats [32]. In cerebral ischemia, the subacute prophylactic administration of zinc (2.5 mg/kg/4 days) prevented the loss of long-term memory, suggesting the development of neuroplasticity [23].

The antecedents exposed here validate the neuroprotective effect of the acute and subacute administration of zinc. However, the effect of chronic administration of zinc is controversial in other animal models and remains unknown during cerebral ischemia. In support of the hazardous effects of zinc, the chronic administration of ZnO (3.5 mg/m^3^, each 4 h/day) inhaled during a period of 13 weeks caused inflammation, cytotoxicity, and histopathological changes in the lungs [33]. In addition, chronic administration of zinc decreased the concentration of magnesium and copper and increased sodium concentration in the brain tissue, consequently improving the permeability of the blood brain barrier [34]. In support of the neuroprotective effect, chronic zinc administration (zinc hydroaspartate, 10 and 65 mg/kg/14 days) exhibits antidepressant activity by increasing the presynaptic zinc concentration in the prefrontal cortex in the rat [35].

This work aimed to clarify whether the prophylactic chronic administration of zinc (ZnCl_2_) at tolerable doses (0.5 mg/kg every 24 h for 14 days) known to produce zinc accumulation in the hippocampus will cause neurotoxicity or neuroprotection in a cerebral hypoxia-ischemia rat model. Thus, we evaluated nitrosative stress, inflammatory process, and cell death (hematoxylin and eosin staining) in the cerebral cortex-hippocampus after 10-minute common carotid artery occlusion (CCAO). We selected 84 genes coding for cytokines, chemokines, and their receptors as well as other related genes, in order to identify which of them exhibit altered expression due to the chronic prophylactic administration of zinc during cerebral ischemia. This issue is still unknown and its clarification will provide new insight into the neuroinflammatory response as to those chemokines and their receptors. Morris water maze test was used to assess whether the chronic administration of zinc prevents the CCAO-induced memory loss. Our results suggest that the chronic administration of zinc at tolerable doses does not have a protective effect in an animal model of cerebral hypoxia-ischemia.

## 2. Materials and Methods

### 2.1. Experimental Animals

Male Wistar rats (body weight 190 to 240 g) were obtained from the vivarium of CINVESTAV and maintained in adequate rooms with controlled conditions for temperature (22 ± 3°C) and a light-dark cycle (12 h-12 h, light onset at 07:00). Food and water were provided ad libitum. All procedures were in accordance with the Mexican current legislation, the NOM-062-ZOO-1999 (SAGARPA), based on the* Guide for the Care and Use of Laboratory Animals*, NRC. The Institutional Animal Care and Use Committee approved the experimental procedures with protocol number 09-102. All efforts were made to minimize animal suffering.

### 2.2. Zinc Administration

**Figure 1:**
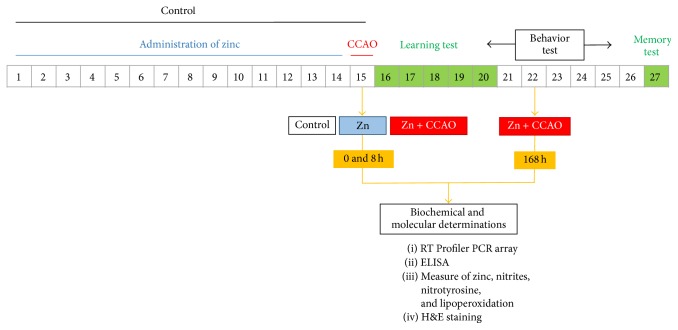
Experimental design of the chronic administration of zinc, performances of the learning test, and determinations of biochemical and molecular variables in Wistar rats.

Rats were grouped as follows: (1) CZn15 d, control rats treated with chronic administration of zinc (ZnCl_2_, 0.5 mg/kg every 24 h for 14 days); their brains were obtained on day 15; (2) Zn15 d+CCAO, rats with chronic administration of zinc that were subjected to CCAO 24 h after the last administration of zinc; their brains were obtained at 8 hours and 7 days after reperfusion; and (3) control, intact rats that did not receive surgery and zinc administration.  Figure 1 shows the animal groups and the experimental design.

### 2.3. Atomic Absorption Spectrometry

Zinc content in temporoparietal-hippocampus tissue was measured using a Varian AA 55B atomic absorption spectrophotometer (Varian S.A., Mexico). Tissues (100 mg) were digested (1 : 1 w/v) with concentrated nitric acid and 70% perchloric acid (1 : 1) for 2 days in prewashed and dried polypropylene tubes at room temperature (RT). The calibration curve of 0.1 to 1 ppm of zinc was made. Zinc concentration in these samples was measured in triplicate and absorbance was determined at 213.9 nm [36].

### 2.4. Nitrites

The temporoparietal cortex and hippocampus of all studied groups (*n* = 5 in each group) were mechanically homogenized in phosphate-buffered saline solution (PBS), pH 7.4, and centrifuged at 12,500 rpm for 30 min at 4°C by using a 17TR microcentrifuge (Hanil Science Industrial Co. Ltd., Incheon, Korea). The NO production was assessed by the accumulation of nitrites (NO_2_^−^) in the supernatants of homogenates, as described elsewhere [37]. Briefly, the nitrite concentration in 100 *μ*L of supernatant was measured by using a colorimetric reaction generated by the addition of 100 *μ*L of Griess reagent, composed of equal volumes of 0.1% N-(1-naphthyl)ethylenediamine dihydrochloride and 1.32% sulfanilamide in 60% acetic acid. The absorbance of the samples was determined at 540 nm with a SmartSpec 3000 spectrophotometer (Bio-Rad, Hercules, CA, USA) and interpolated by using a standard curve of NaNO_2_ (1 to 10 *μ*M) to calculate the nitrite content.

### 2.5. Lipoperoxidation

Malondialdehyde (MDA) and 4-hydroxyalkenals (4-HAD) were measured in supernatants of cerebral cortex-hippocampus homogenates following the method described elsewhere [38]. The colorimetric reaction was made using 200 *μ*L of supernatant after the subsequent addition of 650 *μ*L of 10.3 mM N-methyl-2-phenyl-indole diluted in a mixture of acetonitrile : methanol (3 : 1) and 150 *μ*L of methanesulfonic acid. The reaction mixture was vortexed and incubated at 45°C for 1 h and afterwards centrifuged at 3000 rpm for 10 min. The absorbance in the supernatant was read at 586 nm with a SmartSpec 3000 spectrophotometer (Bio-Rad, Hercules, CA, USA). The absorbance values were compared to a standard curve in the concentration range of 0.5 to 5 *μ*M of 1,1,3,3-tetramethoxypropane (10 mM stock) to calculate the content of malondialdehyde + 4-hydroxyalkenal (MDA + 4-HAD) in the samples.

### 2.6. Enzyme-Linked Immunosorbent Assay (ELISA)

Nitrotyrosine, CCL2/CCR2, CCL4/CCR8, CCL5/CCR5, CXCL1/CXCR2, CXCL12/CXCR4, CXCL13/CXCR5, FGF2, I*κ*B, and NF*κ*B levels were measured by ELISA in homogenates of temporoparietal cortex-hippocampus (*n* = 5 for each group), as described previously [39]. Protein content was determined using Sedmak and Grossberg method [40]. Aliquots containing 5 *μ*g of total protein were placed into wells of ELISA plates. Subsequently, 100 *μ*L of 0.1 M carbonate buffer was added to each well and the plates were incubated at 4°C for 18 h. To block nonspecific binding sites, 200 *μ*L of 0.5% bovine serum albumin (IgG-free) was added to each well at RT. After 30-minute incubation, the wells were washed thrice with phosphate-buffered saline (PBS)-Tween 20 (0.1%) solution. The primary antibodies were rabbit polyclonal anti-nitrotyrosine (1 : 1000, Cat. # N0409, Sigma) and rabbit monoclonal antibodies to CCL2 (1 : 500 dilution; Cat. # AAR31, AbD Serotec, North Carolina, USA), and the following antibodies were obtained from Abcam, San Francisco, USA: CCR2 (1 : 500 dilution; Cat. # ab21667), CCL4 (1 : 500 dilution; Cat. # ab25129), CCL5 (1 : 500 dilution; Cat. # aar18G), CCR5 (1 : 500 dilution; Cat. # ab65850), CCR1 (1 : 500 dilution; Cat. # ab117416), CXCL12 (1 : 500 dilution; Cat. # ab25118), CXCL13 (1 : 500 dilution; Cat. # ab112521), CXCR4 (1 : 500 dilution; Cat. # ab2074), CXCR5 (1 : 500 dilution; Cat. # ab133706), FGF2 (1 : 500 dilution; Cat. # ab106245), and NF*κ*B p65 (phosphor S536, ab86299) and a mouse monoclonal antibody to I*κ*B alpha (1 : 500 dilution; phospho S32 + S36, ab12135) and rabbit polyclonal anti-CCR8 (1 : 500 dilution; Cat. # sc30033, Santa Cruz Biotechnology Inc., Dallas, Texas, USA). The primary antibodies were added to each well and incubated for 2 h at room temperature. After three washes with PBS, horseradish-peroxidase conjugated goat anti-rabbit or mouse IgG (1 : 1000 dilution; Dako North America Inc., Carpinteria, CA, USA) was added to the wells and incubated for 2 h at RT. The antibody-antigen complex was revealed by addition of 100 *μ*L of 2,2′-azinobis(3-ethylbenzthiazoline-6-sulphonic acid) (ABTS) containing 0.3% H_2_O_2_ to each well. After 15 min, optical density (OD) was determined using a benchmark multiplate reader at 415 nm (Bio-Rad, Hercules, CA, USA). All samples were processed under the same experimental conditions and time.

### 2.7. Hematoxylin and Eosin Staining

The morphology changes were analyzed by the histopathology study of the temporoparietal cortex and the hippocampus from the brains of each experimental group was analyzed in coronary brain slices by hematoxylin-eosin staining at 7 days after reperfusion (*n* = 3 in each group). The 3 *μ*m paraffin-embedded tissue sections were stained with hematoxylin and eosin and examined at a magnification objective of 40x (Mod BM 1000, Leica, Jenoptik Camera, Wetzlar, Germany), which provided evidence of cell death [41]. Digital micrographs were made from 5 randomly selected fields of each tissue section of each experimental group (ProgRes CapturePro 2.1, Leica).

### 2.8. PCR Array

Total RNA (1.0 *μ*g) was extracted with TRIzol and quantified using NanoDrop Spectrophotometer (Thermo Scientific NanoDrop Technologies, Wilmington, DE, USA). Reverse transcription reaction was prepared with the RT^2^ PCR array first strand kit from SABiosciences (Qiagen Company). Real-time PCR was conducted to evaluate 84 genes for chemokines and receptors RT^2^ Profiler PCR array of rat (PARN-022Z, Qiagen).

The amplification assays were made using 7900HT Fast Real-Time PCR System (Applied Biosystems, Foster City, CA, USA) (http://www.sabiosciences.com/PCRArrayPlate.php).

### 2.9. Spatial Reference Learning and Memory

The Morris water maze was used to measure the spatial reference memory. The measurements were conducted in a round tank, 150 cm in diameter and 80 cm deep, filled with water and divided into four imaginary quadrants. Water was maintained at a temperature of 23 ± 2°C and dyed white with a titanium dioxide suspension to prevent the rats from locating the platform visually. Several distal visual cues were placed on both walls of the Morris water maze and the room in which it had been installed. This evaluation consisted of four test days with four consecutive trials per day. During the trial, each animal was left in the tank facing the wall and allowed to swim freely to an escape platform (40 cm in height and 15 cm in diameter), which was submerged 2 cm under the water surface and conserved to the center of the southeast (SE) quadrant of the tank. Rats were left in the tank on each of the four vertices of the imaginary quadrants. If the animals did not find the platform during a period of 60 s in the first trial of each test day, they were gently guided to it, allowed to remain on the platform for 30 s, and then removed from the tank. This procedure was used to ensure that the animals retained the visuospatial information of the maze online during the execution of the swimming task [42]. Long-term memory was evaluated in the absence of the platform on day 7 after learning. The latency to reach the platform and the number of times that rats pass by the platform location were measured.

### 2.10. Statistical Analysis

All values are expressed as the mean ± standard error of the mean (SEM) except for the qPCR values which were expressed as a fold change based on a web-based PCR array data analysis protocol (http://pcrdataanalysis.sabiosciences.com/pcr/arrayanalysis.php) which was provided by SABiosciences (Qiagen, Inc.). The differences of Zn15 d+CCAO and CZn15 d groups with respect to the untreated control were determined using unpaired Student's *t*-test. ELISA values were normalized as percentage of the sample value with respect to untreated control. All statistical analyses were performed using the GraphPad Prism software (GraphPad Software Inc., San Diego, CA, USA). *P* values < 0.05 were considered statistically significant.

## 3. Results

**Figure 2:**
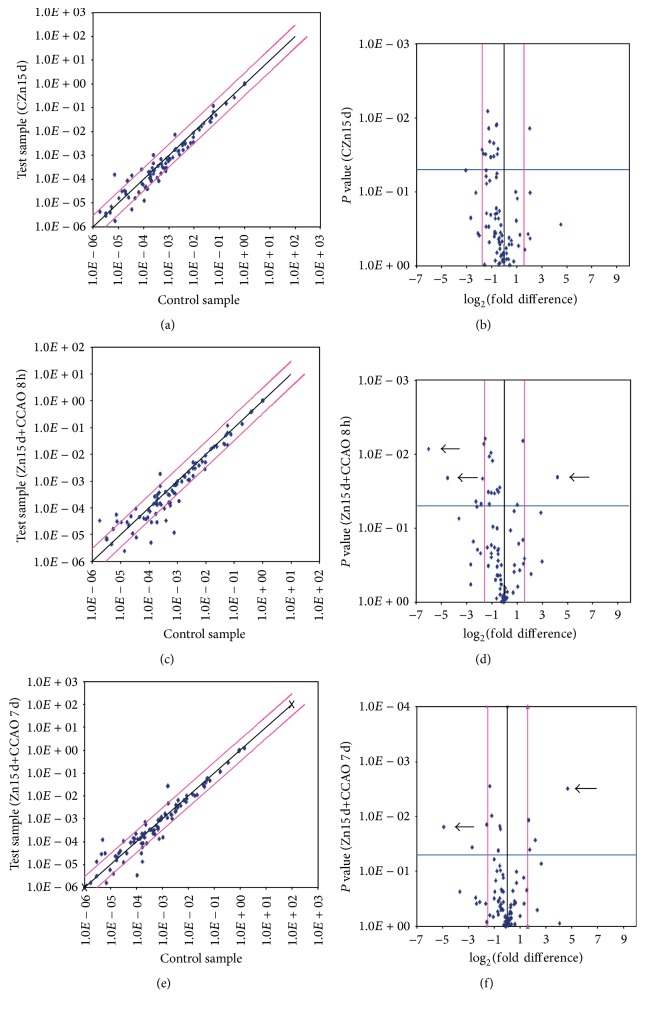
Overview of scatter and volcano plot of expression of 84 genes in temporoparietal cortex-hippocampus using RT^2^ Profiler PCR array. ((a) and (b)) Rats with chronic administration of zinc (CZn15 d). ((c) and (d)) Rats with chronic administration of zinc before CCAO and evaluations at 8 h after reperfusion (Zn15 d+CCAO 8 h). ((e) and (f)) Rats with chronic administration of zinc before CCAO and evaluations at 7 days after reperfusion (Zn15 d+CCAO 7 d). The experimental values were compared with those of untreated rats. The black line indicates fold changes [2^(−ΔCt)^] for scatter plot ((a), (c), and (e)) and log_2_⁡ (fold difference against *P* value) in the volcano plot ((b), (d), and (f)). The pink lines indicate the desired fold change in gene expression threshold. Each value represents the mean of 3 independent experiments. Arrow shows values with high significance.

Chemokines expression profile by qPCR array was analyzed in a scatter and volcano plot (Figure 2). Most values were contained within the gene expression threshold (pink line) in scatter plot of all experimental groups studied (Figures 2(a), 2(c), and 2(e)). The number of transcripts for chemokine and receptors that were downregulated was 6 for the chronic administration of zinc (Figure 2(b)), 12 for 8 h after reperfusion (Figure 2(d)), and 7 for 7 days after reperfusion (Figure 2(f)). The transcripts that were upregulated were 6 for the chronic administration of zinc (Figure 2(b)), 4 for 8 h after reperfusion (Figure 2(d)), and 7 for 7 days after reperfusion (Figure 2(f)).

**Figure 3:**
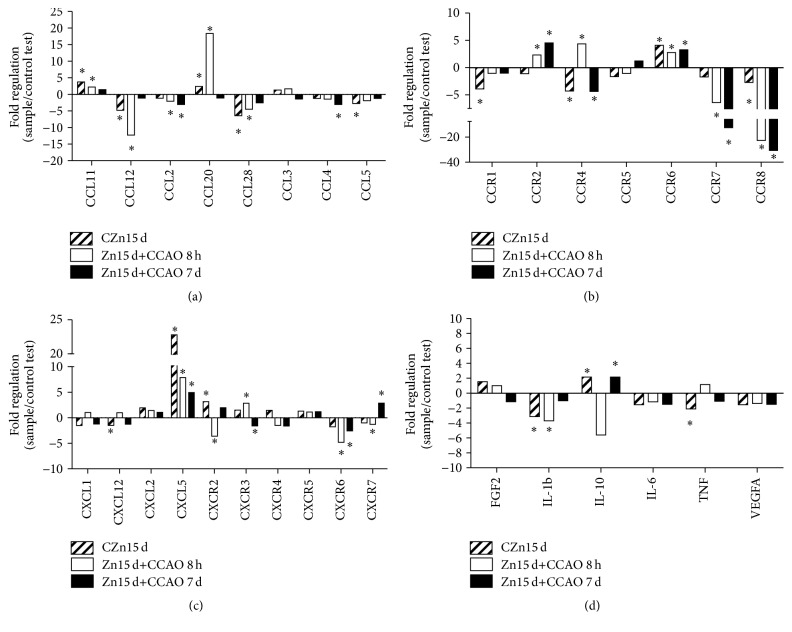
Effect of prophylactic chronic administration of zinc and CCAO on chemokine expression profile in the temporoparietal cortex-hippocampus using RT^2^ Profiler PCR array. CZn15 d, rats with chronic administration of zinc; Zn15 d+CCAO 8 h, rats with chronic administration of zinc before CCAO and evaluations at 8 h after reperfusion; Zn15 d+CCAO 7 d, rats with chronic administration of zinc before CCAO and evaluations at 7 days after reperfusion. Values show fold up- or downregulation as normalized with untreated control values. Each value represents the mean ± SEM of 3 independent experiments performed in triplicate. ^*∗*^Significantly different from untreated rats (Student's *t*-test). The significance was established at *P* < 0.05.

The only prophylactic chronic administration of zinc modified RNA levels for chemokines and receptors compared with untreated control group (Figure 3). The upregulated beta chemokines (Figure 3(a)) were CCL11 (3.75, *P* < 0.05) and CCL20 (2.3879, *P* < 0.05) and those downregulated were CCL12 (−4.77, *P* < 0.05), CCL28 (−6.394, *P* < 0.05), and CCL5 (−2.721, *P* < 0.05). Upregulation of beta chemokine receptor (Figure 3(b)) was established for CCR6 (4.11, *P* < 0.02), whereas downregulation was established for CCR1 (−3.913, *P* < 0.05), CCR4 (−4.286, *P* < 0.05), and CCR8 (−2.712, *P* < 0.05). Upregulation (Figure 3(c)) was established for alpha chemokine CXCL5 (22.8, *P* < 0.05) and alpha chemokine receptor CXCR2 (3.17, *P* < 0.05). Cytokine IL-10 (2.166, *P* < 0.05) was also upregulated (Figure 3(d)), whereas cytokines IL-1b (−3.13, *P* < 0.05) and TNF (−2.104, *P* < 0.05) together with VEGFA (−1.538, *P* < 0.05) were downregulated (Figure 3(d)).

The CCAO modified mRNA levels for chemokines and receptors at 8 h after reperfusion when compared with the untreated control group. The upregulated beta chemokines (Figure 3(a)) were CCL11 (2.22, *P* < 0.05) and CCL20 (18.373, *P* < 0.02), whereas CCL2 (−2.048, *P* < 0.05), CCL5 (−1.893, *P* < 0.05), CCL12 (−12.25, *P* < 0.01), and CCL28 (−4.472, *P* < 0.05) were downregulated (Figure 3(a)). The beta chemokine receptors upregulated (Figure 3(b)) were CCR2 (2.3172, *P* < 0.05), CCR4 (4.3571, *P* < 0.05), and CCR6 (2.7504, *P* < 0.05), and those downregulated were CCR8 (−22.75, *P* < 0.02) and CCR7 (−6.406, *P* < 0.05). Upregulation was established for alpha chemokine CXCL5 (7.8869, *P* < 0.05) and receptor CXCR3 (2.8586, *P* < 0.05) (Figure 3(c)), whereas the downregulated receptors were CXCR2 (−3.607, *P* < 0.05) and CXCR6 (−4.793, *P* < 0.05) (Figure 3(c)). Cytokines that were downregulated (Figure 3(d)) were IL-10 (−5.609, *P* < 0.05) and IL-1b (−3.714, *P* < 0.05).

**Figure 4:**
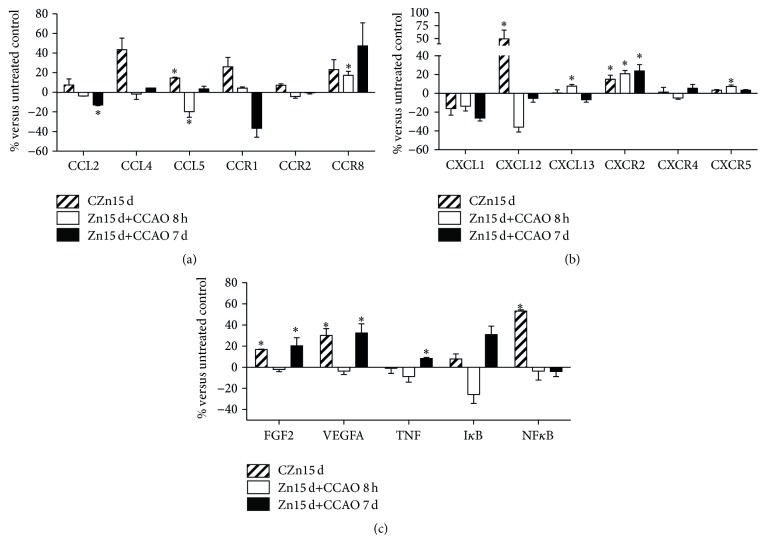
Effect of prophylactic chronic administration of zinc and CCAO on protein levels of chemokines and their receptors in the temporoparietal cortex-hippocampus using ELISA. CZn15 d, rats with chronic administration of zinc; Zn15 d+CCAO 8 h, rats with chronic administration of zinc before CCAO and evaluations at 8 h after reperfusion; Zn15 d+CCAO 7 d, rats with chronic administration of zinc before CCAO and evaluations at 7 days after reperfusion. Experimental values were normalized with respect to those of untreated controls. Each value represents the mean ± SEM of 5 independent experiments made in triplicate. ^*∗*^Significantly different from untreated rats (Student's *t*-test). The significance was established at *P* < 0.05.

The CCAO modified mRNA levels for chemokines and receptors 7 days after reperfusion when compared with the untreated control group (Figure 3). Downregulation was determined for the beta chemokines CCL2 (−3.041, *P* < 0.05), CCL28 (−2.552, *P* < 0.05), and CCL4 (−3.048, *P* < 0.05) (Figure 3(a)). Beta chemokine receptors CCR2 (4.5338, *P* < 0.05) and CCR6 (3.2851, *P* < 0.05) were upregulated (Figure 3(b)), whereas CCR4 (−4.358, *P* < 0.05), CCR7 (−12.54, *P* < 0.05), and CCR8 (−30.6, *P* < 0.02) were downregulated (Figure 3(b)). Upregulation was determined for alpha chemokine CXCL5 (4.95, *P* < 0.05). Alpha chemokine receptors CXCR2 (1.9619, *P* < 0.05) and CXCR7 (2.866, *P* < 0.05) were upregulated (Figure 3(c)), and only CXCR6 (−2.564, *P* < 0.002) was downregulated (Figure 4(c)). Finally, the cytokine IL-10 (2.166, *P* < 0.05) was upregulated as well (Figure 3(d)).

The protein levels of some chemokines and their receptors known to be affected by cerebral ischemia were also evaluated using ELISA and their values were normalized with respect to those of the untreated control group (Figure 4). Prophylactic chronic administration of zinc significantly increased the levels of CCL4, CCL5 (Figure 4(a)), CXCL12, CXCR2 (Figure 4(b)), FGF2, VEGFA, and NF*κ*B (Figure 4(c)). At 8 h after reperfusion, the CCAO considerably increased CCR8 (Figure 4(a)), CXCL13, CXCR2, and CXCR5 levels (Figure 4(b)) and decreased CXCL12 level (Figure 4(b)). On day 7 after reperfusion, the CCAO increased CXCR2 (Figure 4(b)), FGF2, VEGFA, TNF, and I*κ*B (Figure 4(c)).

**Figure 5:**
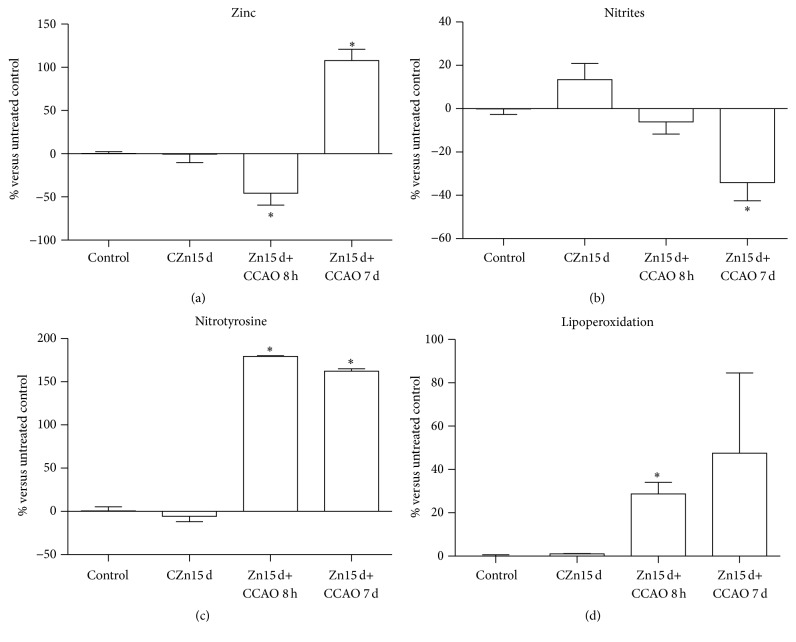
Effect of the chronic prophylactic administration of zinc on levels of zinc and stress nitrosative markers. CZn15 d, rats with chronic administration of zinc; Zn15 d+CCAO 8 h, rats with chronic administration of zinc before CCAO and evaluations at 8 h after reperfusion; Zn15 d+CCAO 7 d, rats with chronic administration of zinc before CCAO and evaluations at 7 days after reperfusion. Values are represented as the percentage with respect to untreated control. Each value represents the mean ± SEM of 5 independent experiments made in triplicate. ^*∗*^Significantly different from untreated rats (Student's *t*-test). The significance was established at *P* < 0.05.

Chronic administration of zinc did not modify the levels of zinc, nitrites, nitrotyrosine, and lipoperoxidation in the temporoparietal-hippocampus of the CZn15 d group when compared with the untreated control group (Figure 5(a)). In rats with chronic treatment of zinc, the CCAO significantly decreased the zinc levels by 46 ± 14% at 8 h after reperfusion and increased the metal levels by 108 ± 13% at 7 days after reperfusion (Figure 5(a)). Nitrite levels were significantly decreased by 34 ± 8% at 7 days after reperfusion (Figure 5(b)), but nitrotyrosine was increased by 179 ± 1% at 8 h and 162 ± 3% at 7 days after reperfusion (Figure 5(c)). Lipoperoxidation increased by 29 ± 5% at 8 h and 7 days after reperfusion when compared with the untreated control group (Figure 5(d)).

**Figure 6:**
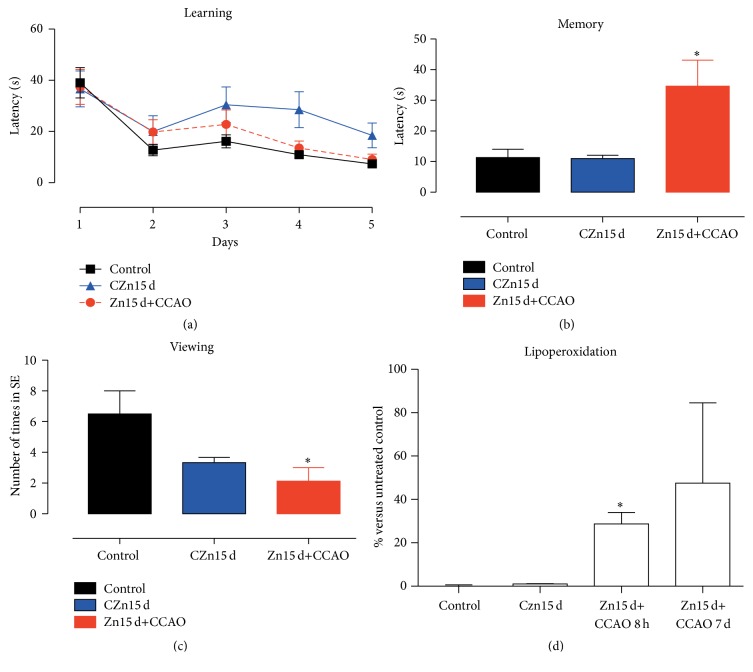
Effect of chronic administration of zinc on learning and long-term memory after hypoxia-ischemia in rats. (a) Graph showing the time course of the latency to reach the escape platform expressed as the mean value of four events of a daily evaluation per rat in the Morris water maze (*n* = 10 rats per group). Graphs showing the long-term memory latency (b) and the number of platform position crossings determined on day 7 after the training, that is, on day 12 after reperfusion (c) (*n* = 5 rats per group). The values are the mean ± SEM. ^*∗*^Significant when compared with the control group; unpaired Student's *t*-test; *P* < 0.05.

To evaluate whether the chronic administration of zinc prevents the CCAO-induced neuronal damage in the hippocampus, spatial reference learning and memory were assessed using Morris water maze. The chronic administration of zinc in the presence or absence of CCAO did not modify the latency of spatial reference learning when compared with the untreated group, suggesting that the learning was maintained on day 5 (Figure 6(a)). CCAO in the rats treated with zinc increased the latency by 34.7 ± 8% on day 7 after the learning training (Figure 6(b)). In addition, the number of times that rats pass by the platform location was decreased by 49% in the rats treated with zinc plus CCAO as compared to the untreated control group (Figure 6(c)), thus suggesting loss of long-term memory.

**Figure 7:**
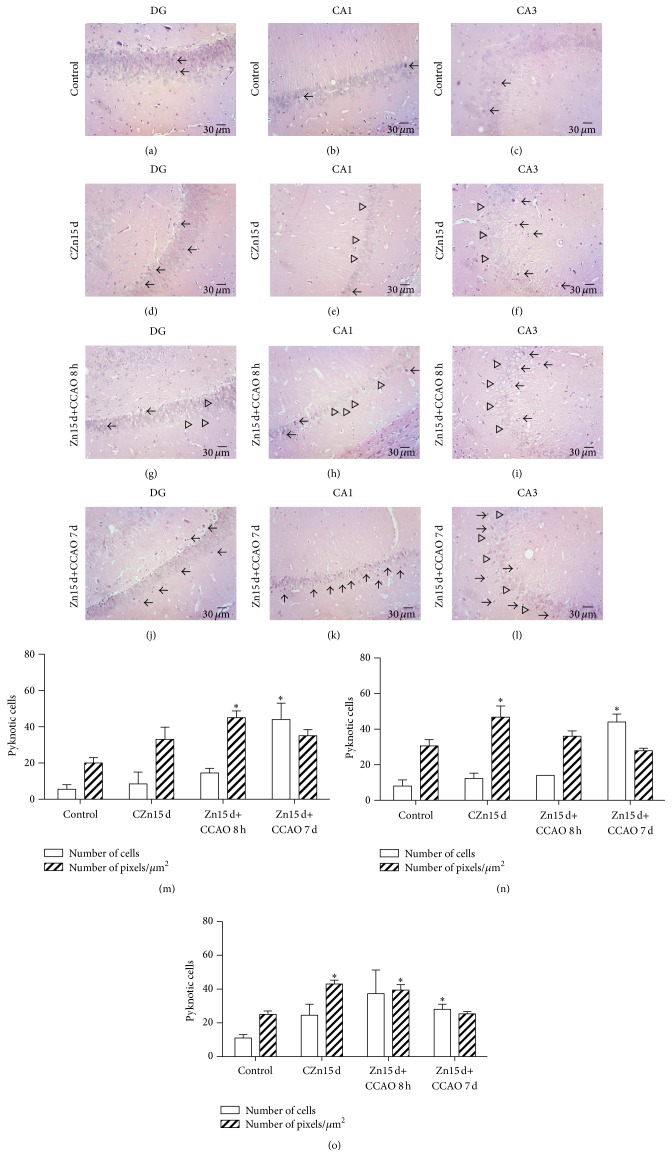
Histopathological evaluation of hippocampus in rats treated with zinc. Paraffin-embedded tissue sections of 3 *μ*m were stained with hematoxylin and eosin. Control, untreated rat; CZn15 d, rats with chronic administration of zinc; Zn15 d+CCAO 8 h, rats with chronic administration of zinc before CCAO and evaluations at 8 h after reperfusion; Zn15 d+CCAO 7 d, rats with chronic administration of zinc before CCAO and evaluations at 7 days after reperfusion. Dentate gyrus ((a), (d), (g), and (j)), CA1 ((b), (e), (h), and (k)), and CA3 ((c), (f), (i), and (l)) regions of the hippocampus. Apoptotic cells (dark arrow); necrosis (clear arrowhead). (((m), DG), ((n), CA1), and ((o), CA3)) Values of cell counts and color intensity (# pixels/*μ*m^2^) using ImageJ, Java-based image processing program. ^*∗*^Significant when compared with the control group; unpaired Student's *t*-test; *P* < 0.05.

The histopathological study showed that the prophylactic chronic administration of zinc caused cell death (black arrow) in the dentate gyrus (DG; Figure 7(j)), CA1 (Figure 7(k)), and CA3 (Figure 7(l)) regions in rats with CCAO mainly on day 7 after reperfusion (Figure 7). The increase in shrinkage and pyknotic cells was 44% ± 9% in DG (Figure 7(m)), 43.5% ± 4.5% in CA1 (Figure 7(n)), and 28% ± 3% in CA3 (Figure 7(o)) regions when compared to the untreated control group, suggesting apoptosis. There was an increase only in intensity in DG (Figure 7(m)) and CA3 (Figure 7(o)) at 8 h after reperfusion and in CA3 of CZn15 d group (Figure 7(o)). The histopathological study also revealed the presence of cells with characteristics of necrosis (edematous cells, pale and ghost cells) in these three regions of hippocampus of CZn15 d group (Figures 7(d)–7(f)). Necrosis (clear arrowhead) was most severe in rats with CCAO at 8 h (Figures 7(g)–7(i)) and 7 days (Figures 7(j)–7(l)) after reperfusion.

## 4. Discussion

Our results show that the prophylactic chronic administration of zinc in rats with CCAO does not prevent the nitrosative stress and neuroinflammation in the early and late phase of cerebral hypoxia-ischemia. The zinc accumulation in the late phase of cerebral hypoxia-ischemia suggests dysregulation of zinc homeostasis because of nitrosative stress.

The development of neuroinflammation is supported by upregulation of CCL11, CCL20, and CXCL5 and receptors such as CCR6 and CXCR2 in rats treated with zinc in the presence or absence of CCAO. These chemokines and receptors are involved in the disruption of the blood brain barrier (BBB), thus promoting leucocyte infiltration. CCL11, which is released by astrocytes, promotes infiltration of marrow-derived cells (eosinophil, monocytes, microglia, and dendritic cells) selectively acting through the CC-motif receptor 3 (Ccr3), to promote cytotoxicity [43–47]. CCL20 that activates CCR6 mediates the direct interaction of T cells with BBB and their attraction towards diencephalon and brainstem [48]. This chemokine is known to play an important role in neuroinflammation induced by the focal cerebral ischemia [49]. CXCL5, produced by microglia/monocytes, is one of the ELR-expressing CXC chemokines and is a potent neutrophil attractant and activator. Increased levels of CXCL5 have been found in the CSF of patients at 24 h after stroke [50]. In addition, this chemokine is a potential biomarker for white matter injury in preterm infants [51]. However, CXCL5 has also been associated with a preconditioning effect through activation of CXCR2, which leads to an increase in CXCL12 expression. The molecular mechanism underpinning this preconditioning effect is that CXCR2 activation increases the expression of miR-223, which inhibits NF*κ*B and subsequently miR-27b levels. The downregulation of miR-27b eliminates the inhibition of CXCL12 expression [52], thus promoting neuroprotection [53–55].

The upregulation of CCR2, CCR4, and CXCR3 at 8 h after reperfusion in the rats with CCAO and those chronically pretreated with zinc gives further support to the development of neuroinflammation. CCL2 and CCR2 have been associated with disruption of BBB and neuroinflammation [56, 57]. However, previous studies have also demonstrated that the subacute administration of zinc before CCAO increases CCL2/CCR2 and promotes neuroprotection [23], which is associated with the ischemic preconditioning and postconditioning effect [58, 59]. In contrast, we found that the prophylactic chronic administration of zinc did not significantly increase the protein levels of CCL2 and CCR2 at two time points used in our study. Therefore, these results cannot support the development of neuroprotection through CCL2. Our suggestion that CCR4 and CXCR3 might participate in neuroinflammation is based on previous reports associating CCR4 with recruitment of white blood cells and production of cytokines after myocardial infarction [60] and correlated CXCR3 with leukocyte accumulation in focal stroke and gliosis [61].

Compared to the untreated control rats, an increase in the protein levels of CCL5, CXCL12, and CXCR5 was observed in control rats with chronic administration of zinc. Since these rats showed absence of damage markers (nitrosylation and lipoperoxidation), we can suggest that the chronic administration of zinc caused an ischemic preconditioning effect. The increase in the chemokines might be through RFLAT-1, a zinc-finger transcription factor, as it occurs in lymphocytes [62]. Increased levels of CCL5, CCL2, CCL3, and CXCL12 found in the early phase of endothelin-1 induced stroke model were not the cause of neurodegeneration [63]. CCL5 is produced from neurons after ischemic stroke to induce the production of neurotrophic factors in peri-infarct areas [64]. CXCL12 is reported to play a critical role in neuroprotection after stroke, by recruitment of endothelial progenitor cells and neuronal progenitor cells in the subventricular zone (SVZ) [54, 65, 66] as well as by promoting proliferation, differentiation, and migration of those progenitor cells [55, 67]. CXCL12 upregulation is associated with preconditioning process and decreasing neuroinflammation after stroke in ischemic-tolerant mice [68]. Another effect of CXCL12 is to modulate synaptic transmission to immature neurons during postischemic cerebral repair [54]. These neuroprotection effects are further supported by the prevention of hypoxic-ischemic brain damage after CXCL12 treatment in mice [69, 70]. However, other studies have associated CCL5 and CXCL12 with risk of stroke in human and mouse atherogenesis [71, 72], promoting cell migration into the brain and induction of cytokines. CXCL12 may influence vascular, astroglial, and neuronal functions via CXCR7 and mediate lymphocyte recruitment in the ischemic areas via CXCR4 after stroke, thus supporting its role in neuroinflammation [73], although it has also been reported that CXCR4 and CXCR7 participate in neurogenesis promoting migration of neural progenitors and bone marrow mesenchymal stem cells [67, 74].

Increased levels of FGF2 and VEGFA in rats treated with chronic administration of zinc in the absence of CCAO also suggest a neuroprotective effect. Zinc could induce the release of FGF2 through the activation of the myeloid zinc-finger 1 (MZF-1), a transcription factor present in astrocytes [24]. FGF2 is involved in the suppression of endoplasmic reticulum stress in ischemic oxidative damage models through the activation of PI3K/Akt and ERK1/2 pathways [75]. In addition, upregulation of FGF2 in the brain after enriched environment enhanced angiogenesis and improved motor function in chronic hypoxic-ischemic brain injury animal model [76]. VEGF is also known to be involved in neuroprotection after ischemia stroke promoting angiogenesis and neurogenesis [77]. In addition, VEGF ameliorates cognitive impairment and synaptic plasticity via improving neuronal viability and function through acting on VEGFR2 [78]. This effect might explain the conservation of spatial memory in the control chronically pretreated with zinc that we found in this study.

However, despite the increase in FGF2 and VEGFA levels, there was no neuroprotective effect at 7 days afer reperfusion, as shown by the loss of spatial memory and an increase in lipoperoxidation and neuronal cell death. At least the increases of VEGFA agree with the association of the risk of incident stroke/transient ischemic attack [79] as well as the occurrence and development of cognitive impairment in stroke [80].

CCR8, CXCL13, and CXCR2 levels were also increased by the chronic zinc pretreatment at 8 h after reperfusion at 7 days after CCAO, without upregulation of their mRNAs. This difference might be explained by either possible posttranscription regulation through miRNAs or the infiltration of leucocytes carrying those chemokine receptors. In addition, increased nitrosylation and lipoperoxidation were observed from 8 h after reperfusion and an excessive zinc accumulation was associated with cellular death, which occurred at 7 days after reperfusion. Association of the increased levels of those cytokines with cerebral damage markers might suggest their participation in neuroinflammation. Of these three chemokines, only CCR8 might participate in neuroinflammation because it has been suggested to play a significant role in the pathogenesis of chronic relapsing experimental autoimmune encephalomyelitis [81] and pilocarpine-induced status epilepticus [82]. CXCR2 does not participate in the ischemia-induced brain injury because CXCR2 antagonists did not improve outcome despite an increase in CXCR2 protein and mRNA levels in microglia cells [83, 84]. On the contrary, CXCL13, a B cell-specific chemokine, might participate in prevention of neurovascular protection from stroke [85].

Chronic administration of zinc showed an opposite effect on NF*κ*B protein levels in the presence and absence of CCAO. The prophylactic zinc treatment increased NF*κ*B protein levels in the absence of CCAO, while decreased NF*κ*B protein levels were observed after CCAO. In the absence of CCAO, NF*κ*B might be involved in the induction of inflammatory chemokines such as CCL2, CCL5, CXCL12, and CXCR2 [84, 86–88], which participate in the preconditioning effect of zinc. Accordingly, there was no increase in damage markers in the control rats subjected to the chronic administration of zinc. A proposal molecular mechanism of NF*κ*B in the preconditioning effect of zinc is the induction of SODI, which decreases the oxidative stress [89] and exerts an anti-inflammatory effect by reducing CCL2 and CCL3 [90]. The decreased NF*κ*B protein levels after CCAO in rats with prophylactic chronic administration might be caused by the excessive accumulation of zinc, which is known to inhibit the activity of NF*κ*B [91].

The neuronal injury in the late phase of CCAO might be caused by an excessive accumulation of zinc in presynaptic vesicles and synaptic space of the hippocampus in rats with prophylactic chronic treatment of zinc as previously shown [35]. This vesicular zinc may be released by nitrosative stress observed at 8 h after reperfusion and as part of the positive feedback cycle between ROS/RNS generation and increased zinc release [92], causing excitotoxicity [93]. This phenomenon can also result in the loss of long-term memory in rats with CCAO and those chronically pretreated with zinc, in contrast with the improvement in the long-term memory caused by the subacute administration of zinc in rats with CCAO [23].

## 5. Conclusion

In summary, our data show that the chronic administration of zinc at tolerable doses causes nitrosative stress-mediated zinc accumulation that leads to cytotoxicity, neuroinflammation, neuronal death, and cerebral dysfunction after CCAO. Although the chronic administration of zinc at tolerable doses exerts a preconditioning effect in treated control rats, such effect was unable to prevent the cerebral damage after CCAO. It would be interesting to explore the ability of lower doses of zinc alone or in combination with antioxidants to maintain the neuroprotection effect against cerebral ischemia.

## References

[1] Johnston S. C., Mendis S., Mathers C. D. (2009). Global variation in stroke burden and mortality: estimates from monitoring, surveillance, and modelling. *The Lancet Neurology*.

[2] Tuttolomondo A., Di Raimondo D., di Sciacca R., Pinto A., Licata G. (2008). Inflammatory cytokines in acute ischemic stroke. *Current Pharmaceutical Design*.

[3] Cuartero M. I., Ballesteros I., Lizasoain I., Moro M. A. (2015). Complexity of the cell-cell interactions in the innate immune response after cerebral ischemia. *Brain Research*.

[4] Mirabelli-Badenier M., Braunersreuther V., Viviani G. L. (2011). CC and CXC chemokines are pivotal mediators of cerebral injury in ischaemic stroke. *Thrombosis and Haemostasis*.

[5] Zlotnik A., Yoshie O. (2000). Chemokines: a new classification system and their role in immunity. *Immunity*.

[6] Gordon R. J., McGregor A. L., Connor B. (2009). Chemokines direct neural progenitor cell migration following striatal cell loss. *Molecular and Cellular Neuroscience*.

[7] Liu T., Young P. R., McDonnell P. C., White R. F., Barone F. C., Feuerstein G. Z. (1993). Cytokine-induced neutrophil chemoattractant mRNA expressed in cerebral ischemia. *Neuroscience Letters*.

[8] Tei N., Tanaka J., Sugimoto K. (2013). Expression of MCP-1 and fractalkine on endothelial cells and astrocytes may contribute to the invasion and migration of brain macrophages in ischemic rat brain lesions. *Journal of Neuroscience Research*.

[9] Yang B., Xi X., Aronowski J., Savitz S. I. (2012). Ischemic stroke may activate bone marrow mononuclear cells to enhance recovery after stroke. *Stem Cells and Development*.

[10] Zhang T., Li P. (2005). Expression and significance of mRNA for MIP-1alpha in cerebral tissue of newborn rat with hypoxic-ischemic brain damage. *Sichuan Da Xue Xue Bao Yi Xue Ban*.

[11] Takami S., Nishikawa H., Minami M. (1997). Induction of macrophage inflammatory protein MIP-1*α* mRNA on glial cells after focal cerebral ischemia in the rat. *Neuroscience Letters*.

[12] Terao S., Yilmaz G., Stokes K. Y. (2008). Blood cell-derived RANTES mediates cerebral microvascular dysfunction, inflammation, and tissue injury after focal ischemia-reperfusion. *Stroke*.

[13] Li M., Hale J. S., Rich J. N., Ransohoff R. M., Lathia J. D. (2012). Chemokine CXCL12 in neurodegenerative diseases: an SOS signal for stem cell-based repair. *Trends in Neurosciences*.

[14] Yoshida E., Atkinson T. G., Chakravarthy B. (2004). Neuroprotective gene expression profiles in ischemic cortical cultures preconditioned with IGF-1 or bFGF. *Molecular Brain Research*.

[15] Shi W.-Q., Zheng G.-Y., Chen X.-D., Zhu Y.-G., Zhang J., Jiang Q. (2013). The expression of bFGF, GAP-43 and neurogenesis after cerebral ischemia/reperfusion in rats. *Zhongguo Ying Yong Sheng Li Xue Za Zhi*.

[16] Haase H., Rink L. (2014). Multiple impacts of zinc on immune function. *Metallomics: Integrated Biometal Science*.

[17] Haase H., Rink L. (2014). Zinc signals and immune function. *BioFactors*.

[18] Foster M., Samman S. (2012). Zinc and Regulation of inflammatory cytokines: implications for cardiometabolic disease. *Nutrients*.

[19] Rink L., Haase H. (2007). Zinc homeostasis and immunity. *Trends in Immunology*.

[20] Mayer L. S., Uciechowski P., Meyer S., Schwerdtle T., Rink L., Haase H. (2014). Differential impact of zinc deficiency on phagocytosis, oxidative burst, and production of pro-inflammatory cytokines by human monocytes. *Metallomics*.

[21] Yamasaki Y., Suzuki T., Yamaya H., Matsuura N., Onodera H., Kogure K. (1992). Possible involvement of interleukin-1 in ischemic brain edema formation. *Neuroscience Letters*.

[22] Blanco-Alvarez V. M., Lopez-Moreno P., Soto-Rodriguez G. (2013). Subacute zinc administration and L-NAME caused an increase of NO, Zinc, lipoperoxidation, and caspase-3 during a cerebral hypoxia-ischemia process in the rat. *Oxidative Medicine and Cellular Longevity*.

[23] Blanco-Alvarez V. M., Soto-Rodriguez G., Gonzalez-Barrios J. A. (2015). Prophylactic Subacute administration of zinc increases CCL2, CCR2, FGF2, and IGF-1 expression and prevents the long-term memory loss in a rat model of cerebral hypoxia-ischemia. *Neural Plasticity*.

[24] Luo X., Zhang X., Shao W., Yin Y., Zhou J. (2009). Crucial roles of MZF-1 in the transcriptional regulation of apomorphine-induced modulation of FGF-2 expression in astrocytic cultures. *Journal of Neurochemistry*.

[25] Yamamoto C., Fukuda N., Matsumoto T., Higuchi T., Ueno T., Matsumoto K. (2010). Zinc-finger transcriptional factor Sall1 induces angiogenesis by activation of the gene for VEGF-A. *Hypertension Research*.

[26] MacDonald R. S. (2000). The role of zinc in growth and cell proliferation. *Journal of Nutrition*.

[27] Zhao G.-H., Yu P., Hu X.-S., Zhao L. (2004). Effect of Zn(II) on the structure and biological activity of natural *β*-NGF. *Acta Biochimica et Biophysica Sinica*.

[28] Vogt K., Mellor J., Tong G., Nicoll R. (2000). The actions of synaptically released zinc at hippocampal mossy fiber synapses. *Neuron*.

[29] Ketterman J. K., Li Y. V. (2008). Presynaptic evidence for zinc release at the mossy fiber synapse of rat hippocampus. *Journal of Neuroscience Research*.

[30] Palmer T. D., Willhoite A. R., Gage F. H. (2000). Vascular niche for adult hippocampal neurogenesis. *The Journal of Comparative Neurology*.

[31] Yang Y., Jing X.-P., Zhang S.-P. (2013). High dose zinc supplementation induces hippocampal zinc deficiency and memory impairment with inhibition of BDNF signaling. *PLoS ONE*.

[32] Piechal A., Blecharz-Klin K., Pyrzanowska J., Widy-Tyszkiewicz E. (2012). Maternal zinc supplementation improves spatial memory in rat pups. *Biological Trace Element Research*.

[33] Adamcakova-Dodd A., Stebounova L. V., Kim J. S. (2014). Toxicity assessment of zinc oxide nanoparticles using sub-acute and sub-chronic murine inhalation models. *Particle and Fibre Toxicology*.

[34] Yorulmaz H., Şeker F. B., Demir G., Yalçin I. E., Öztaş B. (2013). The effects of zinc treatment on the blood-brain barrier permeability and brain element levels during convulsions. *Biological Trace Element Research*.

[35] Sowa-Kućma M., Kowalska M., Szlósarczyk M. (2011). Chronic treatment with zinc and antidepressants induces enhancement of presynaptic/extracellular zinc concentration in the rat prefrontal cortex. *Amino Acids*.

[36] Brambila E., Munoz-Sánchez J. L., Albores A., Waalkes M. (1999). Early effects of surgery on zinc and metallothionein levels in female rats. *Biological Trace Element Research*.

[37] León-Chávez B. A., Gonzalez-Barrios J. A., Ugarte A., Meraz M. A., Martinez-Fong D. (2003). Evidence in vitro of glial cell priming in the taiep rat. *Brain Research*.

[38] Gerard-Monnier D., Erdelmeier I., Regnard K., Moze-Henry N., Yadan J. C., Chaudiere J. (1998). Reactions of 1-methyl-2-phenylindole with malondialdehyde and 4-hydroxyalkenals. Analytical applications to a colorimetric assay of lipid peroxidation. *Chemical Research in Toxicology*.

[39] Alicia Leon-Chavez B., Aguilar-Alonso P., Antonio Gonzalez-Barrios J. (2006). Increased nitric oxide levels and nitric oxide synthase isoform expression in the cerebellum of the taiep rat during its severe demyelination stage. *Brain Research*.

[40] Sedmak J. J., Grossberg S. E. (1977). A rapid, sensitive, and versatile assay for protein using Coomassie brilliant blue G250. *Analytical Biochemistry*.

[41] Elmore S. (2007). Apoptosis: a review of programmed cell death. *Toxicologic Pathology*.

[42] Morris R. (1984). Developments of a water-maze procedure for studying spatial learning in the rat. *Journal of Neuroscience Methods*.

[43] Erickson M. A., Morofuji Y., Owen J. B., Banks W. A. (2014). Rapid transport of CCL11 across the blood-brain barrier: regional variation and importance of blood cells. *The Journal of Pharmacology and Experimental Therapeutics*.

[44] Beaulieu S., Robbiani D. F., Du X. (2002). Expression of a functional eotaxin (CC chemokine ligand 11) receptor CCR3 by human dendritic cells. *The Journal of Immunology*.

[45] Williams J. L., Holman D. W., Klein R. S. (2014). Chemokines in the balance: maintenance of homeostasis and protection at CNS barriers. *Frontiers in Cellular Neuroscience*.

[46] Warrington J. P. (2015). Placental ischemia increases seizure susceptibility and cerebrospinal fluid cytokines. *Physiological Reports*.

[47] Collington S. J., Westwick J., Williams T. J., Weller C. L. (2010). The function of CCR3 on mouse bone marrow-derived mast cells in vitro. *Immunology*.

[48] Sallusto F., Impellizzieri D., Basso C. (2012). T-cell trafficking in the central nervous system. *Immunological Reviews*.

[49] Terao Y., Ohta H., Oda A., Nakagaito Y., Kiyota Y., Shintani Y. (2009). Macrophage inflammatory protein-3alpha plays a key role in the inflammatory cascade in rat focal cerebral ischemia. *Neuroscience Research*.

[50] Zaremba J., Skrobański P., Losy J. (2006). The level of chemokine CXCL5 in the cerebrospinal fluid is increased during the first 24 hours of ischaemic stroke and correlates with the size of early brain damage. *Folia Morphologica*.

[51] Wang L.-Y., Tu Y.-F., Lin Y.-C., Huang C.-C. (2016). CXCL5 signaling is a shared pathway of neuroinflammation and blood-brain barrier injury contributing to white matter injury in the immature brain. *Journal of Neuroinflammation*.

[52] Shin J. H., Park Y. M., Kim D. H. (2014). Ischemic brain extract increases SDF-1 expression in astrocytes through the CXCR2/miR-223/miR-27b pathway. *Biochimica et Biophysica Acta (BBA)—Gene Regulatory Mechanisms*.

[53] Yoo J., Seo J.-J., Eom J.-H., Hwang D.-Y. (2012). Effects of stromal cell-derived factor 1*α* delivered at different phases of transient focal ischemia in rats. *Neuroscience*.

[54] Ardelt A. A., Bhattacharyya B. J., Belmadani A., Ren D., Miller R. J. (2013). Stromal derived growth factor-1 (CXCL12) modulates synaptic transmission to immature neurons during post-ischemic cerebral repair. *Experimental Neurology*.

[55] Cui L., Qu H., Xiao T., Zhao M., Jolkkonen J., Zhao C. (2013). Stromal cell-derived factor-1 and its receptor CXCR4 in adult neurogenesis after cerebral ischemia. *Restorative Neurology and Neuroscience*.

[56] Ge S., Song L., Serwanski D. R., Kuziel W. A., Pachter J. S. (2008). Transcellular transport of CCL2 across brain microvascular endothelial cells. *Journal of Neurochemistry*.

[57] Dimitrijevic O. B., Stamatovic S. M., Keep R. F., Andjelkovic A. V. (2007). Absence of the chemokine receptor CCR2 protects against cerebral ischemia/reperfusion injury in mice. *Stroke*.

[58] Rehni A. K., Singh T. G. (2012). Involvement of CCR-2 chemokine receptor activation in ischemic preconditioning and postconditioning of brain in mice. *Cytokine*.

[59] Stowe A. M., Wacker B. K., Cravens P. D. (2012). CCL2 upregulation triggers hypoxic preconditioning-induced protection from stroke. *Journal of Neuroinflammation*.

[60] Dong F., Khalil M., Kiedrowski M. (2010). Critical role for leukocyte hypoxia inducible factor-1*α* expression in post-myocardial infarction left ventricular remodeling. *Circulation Research*.

[61] Wang X., Li X., Schmidt D. B. (2000). Identification and molecular characterization of rat CXCR3: receptor expression and interferon-inducible protein-10 binding are increased in focal stroke. *Molecular Pharmacology*.

[62] Song A., Patel A., Thamatrakoln K. (2002). Functional domains and DNA-binding sequences of RFLAT-1/KLF13, a Krüppel-like transcription factor of activated T lymphocytes. *The Journal of Biological Chemistry*.

[63] Wolinski P., Glabinski A. (2013). Chemokines and neurodegeneration in the early stage of experimental ischemic stroke. *Mediators of Inflammation*.

[64] Tokami H., Ago T., Sugimori H. (2013). RANTES has a potential to play a neuroprotective role in an autocrine/paracrine manner after ischemic stroke. *Brain Research*.

[65] Robin A. M., Zhang Z. G., Wang L. (2006). Stromal cell-derived factor 1*α* mediates neural progenitor cell motility after focal cerebral ischemia. *Journal of Cerebral Blood Flow and Metabolism*.

[66] Wu Y., Peng H., Cui M., Whitney N. P., Huang Y., Zheng J. C. (2009). CXCL12 increases human neural progenitor cell proliferation through Akt-1/FOXO3a signaling pathway. *Journal of Neurochemistry*.

[67] Merino J. J., Bellver-Landete V., Oset-Gasque M. J., Cubelos B. (2015). CXCR4/CXCR7 molecular involvement in neuronal and neural progenitor migration: focus in CNS repair. *Journal of Cellular Physiology*.

[68] Selvaraj U. M., Ortega S. B., Hu R. (2016). Preconditioning-induced CXCL12 upregulation minimizes leukocyte infiltration after stroke in ischemia-tolerant mice. *Journal of Cerebral Blood Flow & Metabolism*.

[69] Yu Q., Zhou L., Liu L. (2015). Stromal cell-derived factor-1 alpha alleviates hypoxic-ischemic brain damage in mice. *Biochemical and Biophysical Research Communications*.

[70] Li Y., Tang G., Liu Y. (2015). CXCL12 gene therapy ameliorates ischemia-induced white matter injury in mouse brain. *Stem Cells Translational Medicine*.

[71] Shahrara S., Park C. C., Temkin V., Jarvis J. W., Volin M. V., Pope R. M. (2006). RANTES modulates TLR4-induced cytokine secretion in human peripheral blood monocytes. *The Journal of Immunology*.

[72] Abi-Younes S., Sauty A., Mach F., Sukhova G. K., Libby P., Luster A. D. (2000). The stromal cell-derived factor-1 chemokine is a potent platelet agonist highly expressed in atherosclerotic plaques. *Circulation Research*.

[73] Schönemeier B., Schulz S., Hoellt V., Stumm R. (2008). Enhanced expression of the CXCl12/SDF-1 chemokine receptor CXCR7 after cerebral ischemia in the rat brain. *Journal of Neuroimmunology*.

[74] Wang Y., Fu W., Zhang S. (2014). CXCR-7 receptor promotes SDF-1*α*-induced migration of bone marrow mesenchymal stem cells in the transient cerebral ischemia/reperfusion rat hippocampus. *Brain Research*.

[75] Wang L., Chen Q., Li G., Ke D. (2012). Ghrelin stimulates angiogenesis via GHSR1a-dependent MEK/ERK and PI3K/Akt signal pathways in rat cardiac microvascular endothelial cells. *Peptides*.

[76] Seo J. H., Yu J. H., Suh H., Kim M.-S., Cho S.-R. (2013). Fibroblast growth factor-2 induced by enriched environment enhances angiogenesis and motor function in chronic hypoxic-ischemic brain injury. *PLoS ONE*.

[77] Ruan Q., Zhao C., Ye Z., Ruan J., Xie Q., Xie W. (2015). Effect and possible mechanism of monocyte-derived VEGF on monocyte-endothelial cellular adhesion after electrical burns. *Burns*.

[78] Yang J., Yao Y., Chen T., Zhang T. (2014). VEGF ameliorates cognitive impairment in in vivo and in vitro ischemia via improving neuronal viability and function. *NeuroMolecular Medicine*.

[79] Pikula A., Beiser A. S., Chen T. C. (2013). Serum brain-derived neurotrophic factor and vascular endothelial growth factor levels are associated with risk of stroke and vascular brain injury framingham study. *Stroke*.

[80] Ke X.-J., Zhang J.-J. (2013). Changes in HIF-1*α*, VEGF, NGF and BDNF levels in cerebrospinal fluid and their relationship with cognitive impairment in patients with cerebral infarction. *Journal of Huazhong University of Science and Technology: Medical Sciences*.

[81] Bielecki B., Mazurek A., Wolinski P., Glabinski A. (2007). Expression of chemokine receptors CCR7 and CCR8 in the CNS during ChREAE. *Scandinavian Journal of Immunology*.

[82] Liu J. X., Cao X., Tang Y. C., Liu Y., Tang F. R. (2007). CCR7, CCR8, CCR9 and CCR10 in the mouse hippocampal CA1 area and the dentate gyrus during and after pilocarpine-induced status epilepticus. *Journal of Neurochemistry*.

[83] Brait V. H., Rivera J., Broughton B. R. S., Lee S., Drummond G. R., Sobey C. G. (2011). Chemokine-related gene expression in the brain following ischemic stroke: no role for CXCR2 in outcome. *Brain Research*.

[84] Popivanova B. K., Koike K., Tonchev A. B. (2003). Accumulation of microglial cells expressing ELR motif-positive CXC chemokines and their receptor CXCR2 in monkey hippocampus after ischemia-reperfusion. *Brain Research*.

[85] Monson N. L., Ortega S. B., Ireland S. J. (2014). Repetitive hypoxic preconditioning induces an immunosuppressed B cell phenotype during endogenous protection from stroke. *Journal of Neuroinflammation*.

[86] Thompson W. L., Van Eldik L. J. (2009). Inflammatory cytokines stimulate the chemokines CCL2/MCP-1 and CCL7/MCP-3 through NF*κ*B and MAPK dependent pathways in rat astrocytes. *Brain Research*.

[87] Repeke C. E., Ferreira S. B., Claudino M. (2010). Evidences of the cooperative role of the chemokines CCL3, CCL4 and CCL5 and its receptors CCR1+ and CCR5+ in RANKL+ cell migration throughout experimental periodontitis in mice. *Bone*.

[88] Li M., Ransohoff R. M. (2008). Multiple roles of chemokine CXCL12 in the central nervous system: a migration from immunology to neurobiology. *Progress in Neurobiology*.

[89] Song Y. S., Lee Y.-S., Narasimhan P., Chan P. H. (2007). Reduced oxidative stress promotes NF-*κ*B-mediated neuroprotective gene expression after transient focal cerebral ischemia: lymphocytotrophic cytokines and antiapoptotic factors. *Journal of Cerebral Blood Flow & Metabolism*.

[90] Nishi T., Maier C. M., Hayashi T., Saito A., Chan P. H. (2005). Superoxide dismutase 1 overexpression reduces MCP-1 and MIP-1*α* expression after transient focal cerebral ischemia. *Journal of Cerebral Blood Flow and Metabolism*.

[91] Kim M. H., Jeong H. J. (2015). Zinc oxide nanoparticles suppress LPS-induced NF-*κ*B activation by Inducing A20, a negative regulator of NF-*κ*B, in RAW 264.7 macrophages. *Journal of Nanoscience and Nanotechnology*.

[92] Stork C. J., Li Y. V. (2016). Elevated cytoplasmic free zinc and increased reactive oxygen species generation in the context of brain injury. *Acta Neurochirurgica. Supplement*.

[93] Wang W.-M., Liu Z., Liu A.-J. (2015). The Zinc Ion chelating agent TPEN attenuates neuronal death/apoptosis caused by hypoxia/ischemia via mediating the pathophysiological cascade including excitotoxicity, oxidative stress, and inflammation. *CNS Neuroscience and Therapeutics*.

